# Supplementary material for: A Humanized Yeast Phenomic Model of Deoxycytidine Kinase to Predict Genetic Buffering of Nucleoside Analog Cytotoxicity
Source: Genes (Basel). 2019 Sep 30;10(10):770. doi: 10.3390/genes10100770 (PMC6826991; doi:10.3390/genes10100770)
Supplement: Supplementary file 1 [file genes-10-00770-s001.zip › Additional_Files/Additional_File7_GOTermHeatmaps.zip!/Additional_File7_GOTermHeatmaps/proton-transporting_two-sector_ATPase_complex,_catalytic_domain.pdf]

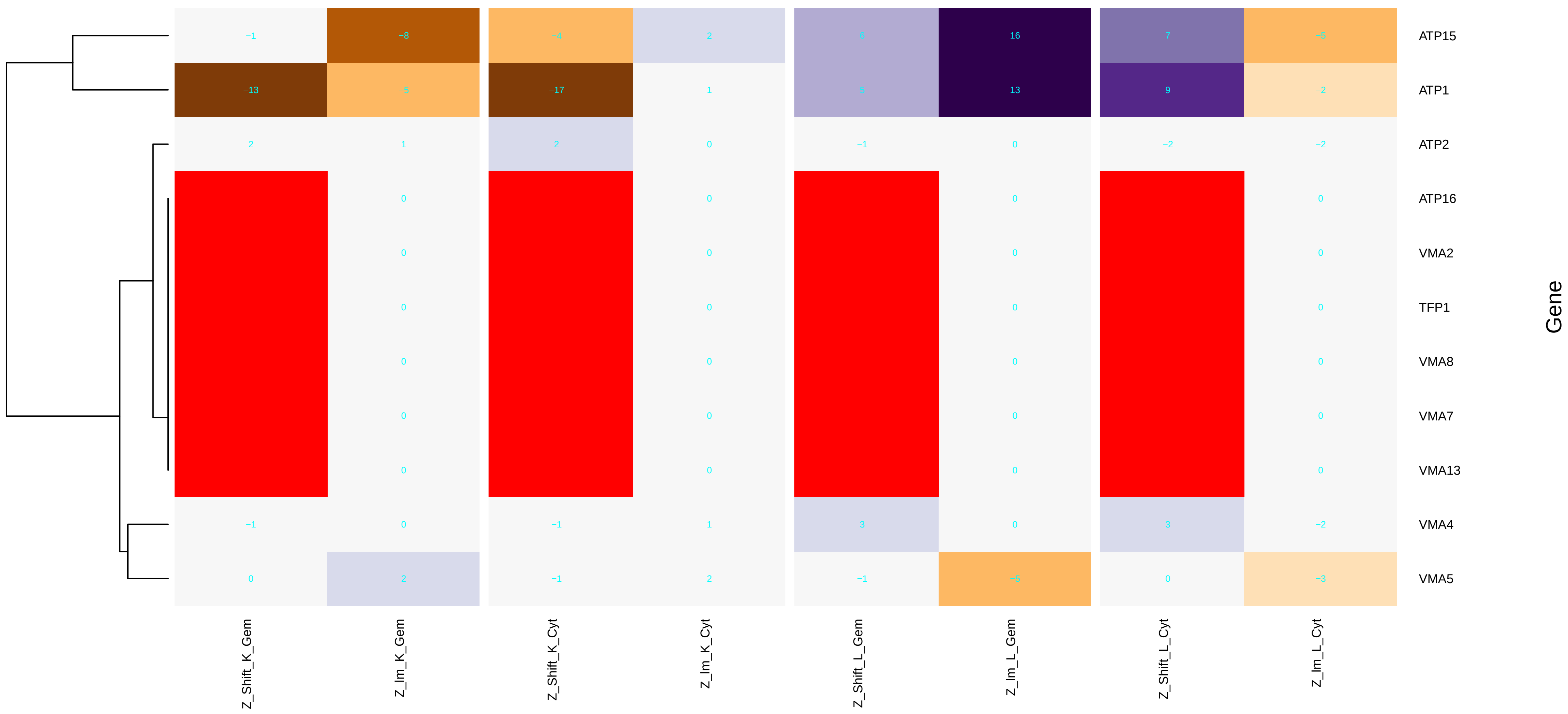
ATP15
−1
−8
−4
2
6
16
7
−5
ATP1
−13
−5
−17
1
5
13
9
−2
ATP2
2
1
2
0
−1
0
−2
−2
ATP16
0
0
0
0
VMA2
0
0
0
0
TFP1
0
0
0
0
VMA8
0
0
0
0
VMA7
0
0
0
0
VMA13
0
0
0
0
VMA4
−1
0
−1
1
3
0
3
−2
VMA5
0
2
−1
2
−1
−5
0
−3
Gene
Z_Shift_K_Gem
Z_Im_K_Gem
Z_Shift_K_Cyt
Z_Im_K_Cyt
Z_Shift_L_Gem
Z_Im_L_Gem
Z_Shift_L_Cyt
Z_Im_L_Cyt
